# COVID-19 loss of taste and smell: potential psychological repercussions

**DOI:** 10.11604/pamj.2022.43.38.31329

**Published:** 2022-09-21

**Authors:** Nismat Javed, Zainab Ijaz, Ali Hamza Khair, Aimen Asim Dar, Erick Daniel Lopez, Ramsha Abbas, Abu Baker Sheikh

**Affiliations:** 1 Shifa College of Medicine, Shifa Tameer-e-Millat University, Islamabad, Pakistan,; 2 Department of Psychiatry, William Beaumont Hospital, Royal Oak, Michigan, USA,; 3 University of New Mexico Health Sciences Center, Department of Internal Medicine, Albuquerque, New Mexico, USA

**Keywords:** Loss of taste, loss of smell, olfactory training, COVID-19, virtual reality

## Abstract

The novel coronavirus (COVID-19) has become a cause for global concern. Apart from a multitude of symptoms, the virus is known for its ability to cause loss of taste and smell that can be irreversible in a few cases. In fact, even after recovery, post-covid syndrome can still lead to devastating outcomes, specifically with reference to loss of smell and taste. A number of mechanisms that have been postulated include receptor-mediated uptake, increased inflammation, transneuronal migration, and direct damage to the olfactory pathway. Considering how important these two senses are, many psychological, social, and emotional repercussions can be expected. These repercussions include lowering of self-esteem and developmental of mental health issues. Long-term altered taste sensation can also lead to the development of unhealthy eating habits that can result in increasing risk for diabetes and hypertension. A few solutions have been investigated for treating these chemosensory dysfunctions, such as olfactory training, corticosteroids, theophylline and acupuncture. Although the results have been promising but a new modality, virtual reality, requires more in-depth exploration because it targets not only the dysfunction but also the mental health issues being experienced. It is important that affected individuals be provided with strong emotional and family support. Additionally, physicians can help the patients through support groups, cognitive behavioural therapy, olfactory, and virtual reality training.

## Introduction

The novel coronavirus (COVID-19) pandemic has currently affected over 161 million people globally, with numbers of death totaling over 3 million at the time of writing (5/14/21). Apart from the typical symptoms, the virus is also notorious for causing olfactory and gustatory dysfunction, observed even in asymptomatic patients, which manifests as hyposmia/anosmia and dysgeusia/hypogeusia/ageusia respectively [1]. Many articles discussing other pandemics, such as influenza, have targeted the psychological aftermath of the global disaster. The term ‘contagion’ is used to describe the result that induces emotional and behavioural changes making individuals more prone to mental health issues. This poses a potential question of consequences that the COVID-19 pandemic can leave behind. This includes chemosensory dysfunction because the chances of consequences are higher with symptoms that are both long-term and irreversible owing to loss of taste and smell. Considering how important these two senses are, many psychological, social, and emotional repercussions can be expected. This review aimed to discuss the prevalence of chemosensory dysfunction, its pathophysiology, potential psychological, social, and emotional repercussions from loss of taste and smell and possible solutions to address these problems.

## Methods

For the purposes of this study, a narrative review was performed. PubMed and Google Scholar were used for retrieval of studies. We reviewed literature from 2019 to 2021. The keywords used were ‘loss of taste’, ‘loss of smell’, ‘olfactory training’, ‘COVID-19’, ‘anosmia’, ageusia’ and ‘virtual reality’. The inclusion criteria for the study were 1) COVID-19 patient population, 2) loss of smell/taste as potential outcomes, 3) prevalence of chemosensory dysfunction, 4) treatment of chemosensory dysfunction and 5) psychological repercussions of loss of taste/smell. Exclusion criteria were studies that did not have the aforementioned specified outcomes or prerequisites, studies not in English language or without an English translation and studies whose data could not be retrieved.

## Current status of knowledge

**Prevalence of loss of smell and taste in COVID-19:** the prevalence of loss of smell and taste varies from 30 to 48% according to some studies [2-4]. Large-scale reviews have reported higher figures. Olfactory dysfunction was noted to be 86.60% (95% CI, 72.95%-95.95%) in one such review [5]. Similar statistics have been reported by a systematic review of studies from many countries, including Europe and Iran with olfactory and gustatory symptoms appearing before general COVID-19 symptoms in 64.5% and 54.0% of the patients, respectively [6]. Multiple studies aimed to determine the exact recovery period from these symptoms. Recovery, although possible within 2 weeks, was commonly observed within a month (77%) [7]. In another study, the loss of smell and taste appeared as the first symptom in 10% and 11% of patients, respectively, and resolved after a mean duration being 9 ± 5 days [8]. In another article, the anosmia and ageusia occurred 4 to 5 days after the appearance of other symptoms of COVID-19 that improved in the first two weeks. However, 34% and 37.5% of the subjects continued to show symptoms for at least 7 days, even after recovery from the disease [9]. A summary of a few studies is shown in Table 1 [2-4]. The chemosensory dysfunction has been linked with the deterioration of the mental health of patients with COVID-19, which includes post-traumatic stress disorder, insomnia, depression, and anxiety. In cases where chemosensory dysfunction is reversible, patients subsequently develop a post-COVID-19 syndrome that makes the course of recovery unpredictable.

**Table 1:** summary of studies reviewed for prevalence

Author	Population	Findings
Saniasiaya *et al*.	Meta-analysis of COVID-19 patients from 2019 to 2020	Prevalence of olfactory dysfunction in COVID-19 patients was 47.85% [95% CI: 41.20-54.50]. 54.40% European, 51.11% North American, 31.39% Asian, and 10.71% Australian COVID-19 patients had the dysfunction
Ibekwe *et al*.	Meta-analysis of COVID-19 patients from 2019 to 2020	Pooled prevalence of loss of smell was 48.47% (95% CI, 33.78%-63.29%). Estimated pooled prevalence of loss of taste was 41.47% (95% CI, 3.13%-31.03%). 13 studies reported combined loss of smell and taste with a pooled prevalence of 35.04% (95% CI, 22.03%-49.26%).
Agyeman *et al*.	Systematic review of patients till 2020	The pooled prevalence of patients presenting with olfactory dysfunction and gustatory dysfunction were 41.0% (95% CI, 28.5% to 53.9%) and 38.2% (95% CI, 24.0% to 53.6%), respectively. Increasing mean age correlated with lower prevalence of olfactory (coefficient = -0.076; P=.02) and gustatory (coefficient = -0.073; P=.03) dysfunctions.

**Pathophysiology of loss of taste and smell in COVID-19:** both ACE2 receptors and TMPRSS2 found in the olfactory and oral epithelial supporting cells can undergo alteration due to the viral damage [10,11]. The virus can transneuronally disseminate into the brain through olfactory pathways and damage the integrity of the neuroepithelium via the same receptors, resulting in anosmia [12-14]. Given these mechanisms, olfactory training could provide rehabilitation by increasing regeneration [14,15]. Direct effect on taste receptors and the inhibition of the retro-nasal pathway might lead to a loss of taste [16]. Because the two senses are related, anosmia could also be the culprit of gustatory dysfunction [10]. Chemosensory disturbances can occur in absence of nasal inflammation and coryzal symptoms [17]. Hence, the virus might be able to target odour processing mechanisms [18]. Brann *et al*. [10], postulated that local infection of the vascular and sustentacular cells in the nasal epithelium could effectively block the conduction of smell [11]. Disruption of cellular water and ionic balances and vascular damage causing hypoperfusion may also be responsible for olfactory bulb dysfunctions [11].

**Previous known viral infections and their association with chemosensory dysfunction:** post-viral olfactory loss has been noted with influenza virus, parainfluenza virus, and rhinovirus [19]. The greatest degree of loss and smell, secondary to respiratory illnesses, was noticed during the onset of coryza [20]. Interestingly, olfactory and gustatory losses were also reported in cases of post-influenza viral syndrome or influenza vaccination. A few predictors such as age, sex, and lifetime infections were associated with vaccination-induced losses of smell and taste (p<0.05) [21,22].

**Figure 1:**
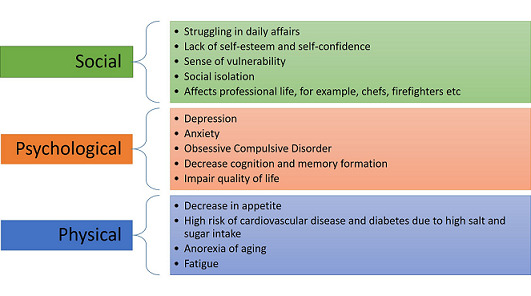
loss of taste and smell due to COVID-19 and its implications

**Psychological impact on patient´s wellbeing with loss of taste and smell:** complete or partial loss of smell or taste attracts less attention and thus, receives no immediate intervention. However, a critical aspect that needs to be investigated is the psychological, emotional as well as social implications of these symptoms if they remain persistent (Figure 1). For example, COVID-19 patients already struggle in their daily affairs due to fatigue and weakness caused by the infection. Sustained changes in olfactory and gustatory function can exacerbate social isolation and cause major changes in their daily activities [23]. This can dramatically impact their self-esteem and overall wellness, making these patients more prone to depression and anxiety.

The association between alteration of these senses and depression is not limited to patients suffering from COVID-19 infection. A systematic review conducted on patients suffering from loss of smell due to various etiologies reported that 40-76% had episodes of depression following the course of olfactory dysfunction [24]. Similar results were reported by another study [25]. Likewise, patients undergoing Trastuzumab therapy for Breast cancer reported feeling worsening in quality of life, as well as a negative impact on social and emotional wellbeing due to reduced taste and smell the following chemotherapy [26].

Both our olfactory and gustatory senses are needed to prepare and enjoy our meals. Diminished or lost senses not only decrease appetite but also lessen social gatherings as people often “socialize over meals” [24]. Long-term dysgeusia can also lead to unhealthy eating habits and thus to various health complications. For example, due to lack of taste, these individuals sometimes increase salt and sugar intake in their foods which increases their risk of cardiovascular disease, diabetes and a lack of healthy balanced diet [27,28]. While older age is associated with other physiological changes such as delayed gastric emptying, a similar association is seen where a decline in sense of smell and taste, as well as depression and loneliness, are thought to contribute to “anorexia of aging.” This often leads to protein malnutrition, frailty, as well as functional deterioration in these patients [29]. Hence, there is a need to identify these problems and intervene early to avoid such complications and social isolation in these patients.

Olfactory function is a vital aspect of our personal life as it is involved in the process of memory formation, social interactions, and personal relationships. A problem that arises with hyposmia and anosmia is the inability to identify body odours, such as from bad breath or sweating. This heightens anxiety in people leading to excessive use of showers as this affects their self-confidence and makes them more vulnerable to a decrease in their social gatherings [28]. This idea can be consolidated by the study conducted on COVID-19 patients, which presented the data of about 76% of the patients reporting a decrease in their quality of life due to loss of smell [30].

The decline in olfactory and gustatory functioning impairs the use of these senses as protective mechanisms and can worsen anxiety in many patients. For example, our olfactory system prevents us from accidents by identifying fires or chemical or gas leaks at our houses. Limiting this ability can create a sense of vulnerability in the patients of not being able to fully prevent themselves or their families from these dangers [24]. Gustatory sense also prevents intake of toxic, burnt, or spoiled food [27]. Moreover, the loss of these senses not only affects our personal life but can also negatively impact our professional life. For instance, people working in the perfume industry or enology, firefighters, chefs, food critics, and many other professions can go through career strain due to a reduction in their senses. This can aggravate anxiety, depression, and disturbances in the mood for these individuals further decreasing their quality of life [28].

**Treatments and rehabilitation for loss of taste and smell:** numerous studies have tested the benefits of systemic exposure to odorants, referred to as olfactory training. Poletti *et al*. carried out a study of olfactory training comparing the outcome of lightweight molecule odours versus heavyweight molecule odours, in which patients with post-viral or post-traumatic olfactory loss benefitted from heavy-weight molecule-based training [31]. Many forms of the therapy have been investigated: change of odour at a fixed time interval, increasing concentration of the odour, and training “balls”. The results showed that all of these therapies were effective (p<0.05) [32-34]. Budesonide irrigation was also investigated as a treatment modality and 43.9% of patients in the budesonide group had improved [35].

Intranasal theophylline was also studied in patients with chemosensory dysfunction. Oral and intranasal treatments had improved both losses of taste and smell. Oral theophylline took a comparatively longer time to act compared to intranasal form (2-12 months vs 1-4 weeks) [36]. Intranasal sodium citrate also demonstrated improvement in olfactory function with sodium citrate, as compared to the control [37]. In a study from 2017, the use of intranasal vitamin A for 8 weeks with the addition of olfactory training had demonstrated 37% improvement in post-infectious patients compared to the control of 23% [38].

Traditional Chinese Acupuncture was introduced to patients not responding to other forms of treatment in a retrospective cohort study. After three months of treatment with multiple acupuncture sites, there was an improvement of smell in about half the patients who underwent acupuncture therapy [39]. In the most recent literature, patients with hyposmia and hypogeusia caused by Major Depressive Disorder underwent a trial of repetitive Transcranial Magnetic Stimulation (tTMS), followed by the Sniffin Sticks and Taste Strips to determine the effects on smell and test. A statistically significant improvement was found in both modalities along with a positive correlation between the odour and smell tests [40].

In a study of 27 subjects with documented COVID-19 infection and persistent olfactory dysfunction, nine participants received both oral corticosteroids that significantly improved their sense of smell (p = 0.007) at the risk of transient side-effects [41]. However, the results from the study might not be applicable long-term because of the increased risk of side effects. Different results have been reported by another trial. Although 62% of the participants recovered their sense of smell by 3 weeks when both the spray and olfactory training were used, this was non-significant (p>0.05) [42].

**Figure 2:**
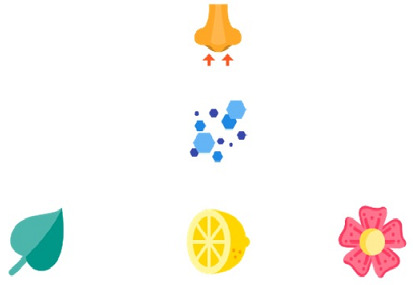
olfactory training consists of sniffing items with high density molecules such as roses, lemons, cloves, or eucalyptus for 20 seconds each, twice a day for ≥3 months; patients who have an altered sense of smell due to COVID-19 infection have shown benefit through the use of this regimen

Recent studies have been investigating the role of visual-olfactory training in patients with anosmia and comparing it to solely olfactory training (Figure 2) [43]. It was also investigated for post-viral rehabilitation. Visual-olfactory training improved the levels of olfactory threshold, discrimination, and identification in another study [44]. Considering the relatively successful impact of the training, a systematic review is currently in progress focusing on COVID-19 induced chemosensory dysfunction [45].

Other studies have shown significant improvement in depression and anxiety through the use of virtual reality [46]. This raises the question of the use of virtual reality as a modality of treatment of COVID-19 Induced Anosmia as well as depression and anxiety. Although patients should be counseled regarding these modalities, virtual reality presents a new dimension that necessitates further investigation to determine if it is a better solution to the problem at hand. A summary of a few studies is presented in Table 2 [31-33].

**Table 2:** summary of studies reviewed for treatment

Author	Population	Findings
Poletti *et al*.	Post-viral or post-traumatic olfactory loss	Olfactory training was associated with olfactory improvement, with a three-time improvement in post-viral olfactory loss patients compared to the post-traumatic group. A greater stimulus (high molecular weight) was associated with more improvement.
Patel *et al*.	Patients with subjective loss of smell and olfactory loss were randomized to olfactory training or control for 6 months.	6/19 patients in the OT group showed significant improvement, while only 2/16 (13%) in the control group improved. Increasing age and duration of loss were significantly correlated to lack of improvement.
Damm *et al*.	A randomized controlled trial with university centers	Olfactory function improved greatly in the high-training group in 18 of 70 participants (26%), compared to the low-training group (15%). In subjects with a duration of olfactory dysfunction of <12 months, olfactory function improved in 15/24 participants (63%) of the high-training group and in 6/31 participants (19%) of the low-training group (P = .03).

**Recommendations:** Primary care providers (PCPs) are some of the frontline health care workers involved in providing support to patients with COVID-19. After the diagnosis of COVID-19, patients can follow up with their PCPs to ensure their overall wellbeing. The role of PCPs regarding care and screening for underlying depression and anxiety from the symptoms, secondary to chemosensory dysfunction, is critical to provide patients optimal care. Important components of managing patients with persistent anosmia and ageusia from COVID-19 that can be provided by their PCPs include: obtaining a thorough history taking, a thorough physical exam including Head, Neck, Eyes, Ears, Nose, and Throat (HEENT), and Cranial Nerves, utilization of assessment tools such as PHQ-2 and if positive then subsequently using PHQ-9, GAD-7, Questionnaire for Olfactory Dysfunction, counseling, and providing them with treatment options.

As COVID-19 has been affecting people globally, more ongoing data has been provided for providers to assist patients who are affected by this virus. The utilization of questionnaires such as PHQ-2, PHQ-9, GAD-7, and Questionnaire for Olfactory Dysfunction can be of benefit to help assist patients being affected by persistent symptoms of anosmia and ageusia from COVID-19. These questionnaires can be done in person or through virtual visits. These have provided benefits to patients as well as resources for mental health.

Individuals affected by the loss of smell and taste require strong emotional support. Family and friends can help during cooking about sugar or salt content as well as provide reassurance regarding body odours, enabling patients to gain self-confidence. Instead of meeting over meals, friends and family can arrange social events that focus more on other activities such as shopping. Work counselors can motivate regarding the changes that the individuals are experiencing in their professional life. Physicians can help the patients by suggesting virtual or in-person groups of people going through similar problems so that they can share their coping mechanisms. Lastly, cognitive behavioural therapy can be used to counter the mental health problems that the patients are experiencing as a consequence of the loss of smell and taste.

## Conclusion

Loss of taste and smell are very important consequences of the COVID-19 infection resulting in mental health issues, particularly depression, anxiety, and low self-esteem. The primary care physicians should screen COVID-19 recovered patients regularly for underlying depression and anxiety, secondary to chemosensory dysfunction. Considering these long-lasting consequences, early screening, diagnosis, and timely interventions are critical in providing patients with optimal care.

### What is known about this topic

- COVID-19 infections are associated with loss of both smell and taste that has known to be irreversible;
- Loss of taste and smell due to COVID-19 are very common symptoms of the infection.

### What this study adds

- Loss of taste and smell are very important consequences of the COVID-19 infection resulting in mental health issues, particularly depression, anxiety, and low self-esteem;
- Primary care providers can play an essential role in screening and managing both the short and long-term complications of the chemosensory dysfunction;
- Virtual reality is a novel treatment modality that should be investigated for reversing this loss of taste and smell.
